# Structural and functional analysis of the TGF-β mimic, TGM-2: an immunomodulatory helminth protein

**DOI:** 10.1038/s41435-025-00372-0

**Published:** 2025-12-18

**Authors:** Emmaculate Yaah Ntang, Kyle T. Cunningham, Shashi P. Singh, Claire Ciancia, Anna Sanders, Sergio Lilla, Ananya Mukundan, Stephen M. Ghogomu, Andrew P. Hinck, Rick M. Maizels

**Affiliations:** 1 School of Infection and Immunity, University of Glasgow, Glasgow, UK; 2 Molecular and Cell Biology Laboratory, University of Buea, Buea, Cameroon; 3 Cancer Research UK Scotland Institute, Glasgow, UK; 4 Department of Structural Biology, University of Pittsburgh, Pittsburgh, PA USA; 5 Present Address: Champalimaud Centre for the Unknown, Lisbon, Portugal; 6 Present Address: Department of Biological Sciences, Birla Institute of Technology and Science, Pilani, Rajasthan India

**Keywords:** Transforming growth factor beta, Genetic interaction

## Abstract

The extraordinary prevalence of helminths is attributable to secretion of molecules that manipulate the host immune system, facilitating their survival. Among the secretory products of the murine intestinal helminth *Heligmosomoides polygyrus* are 10 mimic proteins with functional resemblance to the mammalian immunosuppressive cytokine, TGF-β, but structurally distinct with five Complement Control Protein (CCP) domains, designated TGM-1 to −10. Here we dissect the structure and function of the mimic TGM-2 and its domains. We generated eight protein truncations lacking N- or C-terminal domains, for testing through pulldowns, mass spectrometric analysis and isothermal titration calorimetry, confirming affinity for TGFBR1 (ALK5), TGFBR2, and the co-receptor CD44. We observed that domains 1–3 bind TGFBR1 and TGFBR2, while domains 4 and 5 exhibit stronger binding to the CD44 co-receptor than TGM-1. Additionally, full-length TGM-2 activates the pSMAD pathway in the MFB-F11 fibroblast cell line at concentrations as low as 1 ng/mL and induces the in vitro conversion of naïve murine CD4^+^ T cells into Foxp3^+^ Tregs. Both stimulatory activities diminish significantly in the absence of domains 4 and 5 that interact with CD44. In vivo, both full-length TGM-2 and truncated Domains 1–3 construct potently alleviate allergic airway inflammation in mice exposed to *Alternaria alternata* allergen.

## Introduction

Parasitic helminths have co-evolved with their mammalian host for eons and have developed a profound mastery of the functioning of the host immune system. This mastery enables infectious helminths to manipulate the host immune system to ensure their survival, imposing a significant global burden on both humans and animals, especially in developing countries [1–5]. This burden is exacerbated by their complex life cycles and different ecological niches [6–8], while their success at down-modulating host immunity has been attributed to secretion of a spectrum of immune-suppressive mediators [9–11].

Many studies investigating the intricacies of helminth immune evasion have used the helminth model *Heligmosomoides polygyrus* in laboratory animals [12–14]. For example, Grainger et al. reported that *H. polygyrus* secreted molecules expanded the population of immune modulatory regulatory T cells (Tregs), in a manner dependent on signalling through the TGF-β pathway [15]. Investigation of the secreted molecule(s) responsible for this mechanistic action identified a family of 10 proteins known as *H. polygyrus* TGF-β mimics (TGMs) [16–18].

The first of these to be characterized, TGM-1, functionally mimics the activity of the mammalian immunosuppressive cytokine TGF-β by inducing the expression of Foxp3^+^ Treg cells. TGF-β is known to be highly pivotal for driving immune regulation [19, 20]. However, its activity is tightly regulated as tissue-resident leucocytes produce the dimer in an inactive form linked to a latency-associated peptide [21, 22]. Upon release from the latency-associated protein, proteolytic cleavage yields the mature form of TGF-β, which activates canonical and non-canonical signalling pathways to induce or suppress gene expression [23]. Activation of the canonical TGF-β pathway drives the expression of Foxp3, the key master transcription factor of Tregs.

Although TGF-β induces the expression of Tregs, earlier attempts to generate large numbers of Tregs in vitro for cell therapy to induce immune tolerance revealed the cytokine is limited in its ability to generate Tregs with phenotypic stability [24]. The parasite secreted protein TGM-1, though structurally distinct from the mammalian TGF-β, activates the canonical TGF-β signalling pathway by binding to the serine/threonine kinase type II TGF-β receptor (TGFBR2) which further associates with TGFBR1 [20]. Activated mammalian TGFBR1 recruits and phosphorylates Smad2 and Smad3 which translocate to the nucleus with Smad4 to bind Smad response elements, inducing the expression of genes such as Foxp3. TGM-1 also interacts with CD44 co-receptors for maximal TGF-β activity [25]. Tregs induced when CD4^+^ cells are treated with TGM-1 are phenotypically stable [26], while myeloid cells show reduced pro-inflammatory cytokine expression in response to TGM ligands [27]. Hence, members of the TGM family could be used to treat inflammatory bowel disease [28, 29], allergies [30] and other inflammatory settings [31, 32].

While efforts towards the global eradication of parasitic helminths are currently registering significant success, the discovery of TGMs could be a significant milestone in the exploration of helminth-secreted molecules to treat allergic and autoimmune inflammatory diseases, if their rise in developed countries relates to reduced prevalence of infection as stipulated by the hygiene hypothesis [33]. It is therefore paramount to investigate the structure, function, and mechanism of action of these unique helminth secreted molecules. Compared to TGM-1, TGM-2 is highly similar in overall structure with five complement control protein domains, domains 1-2 (D1-2) which bind TGF-β receptor I (TGFBR1) and are identical between the two proteins, while D3 binding to TGFBR2 and D4-5 to CD44 show significant differences in their amino acid composition [17].

This comparison raises some unanswered questions regarding the exact structure and functions of the domains of the *H. polygyrus* secreted novel TGF-β mimics: Why would the parasite use its most needed energy to make similar proteins? Is TGM-2 in any way more or less potent in mimicking TGF-β activity than TGM-1? How would TGM-2 function without some of its receptor binding domains? In other words, what are the exact functions of these unique receptor binding domains? To answer these questions, we set out to critically analyse the domain structure of TGM-2, identifying its receptor binding properties, and its role in inducing Foxp3^+^ Tregs and reducing innate inflammation in mice.

## Materials and methods

### Mice

BALB/c female mice (purchased from Charles River) and Foxp3-GFP reporter mice (bred-in house from established strain [34]) and aged 6–12 weeks were used for experiments. All animal experiments were performed under UK Home Office licence and approved by the University of Glasgow Animal Welfare and Ethical Review Board, and all procedures were performed in full accordance with relevant guidelines and regulations. Group sizes of 5 were validated by power analysis. No randomization was used, no subjective assessments were used that would have required blinding, and no animals were excluded from analysis.

### Expression of recombinant proteins

*Hp-*TGM-2 from the murine intestinal helminth parasite *Heligmosomoides polygyrus bakeri* has previously been described as a 430-aa full-length protein [17] with sequence deposited under accession number AVN88293. The N-terminal signal peptide sequence of Hp-TGM-2 was predicted by SignalP6.0 to be aa 1–18, and constructs were based on the resulting mature sequence starting from Gly-19. The original sequence was codon-optimized (Supplementary Table 1) and synthesized by Eurofins with flanking *Asc*I/*Not*I restriction digest sites in the 5‘ and 3‘ directions respectively.

The N- and C- truncations of TGM-2 were created by PCR amplification using the Q5 PCR kit from New England Biolabs (ref. E055l), domain-specific primers as shown in Supplementary Tables 2 and 3, and full-length TGM-2 as a template. A 50 µL PCR was carried out with specific conditions previously reported [17]. The resulting PCR products were purified, digested and subcloned into the mammalian expression vector pSecTag2A. Recombinant TGM-2 truncated proteins tagged with histidine were expressed in mammalian Expi293T suspension cell cultures. Overexpressed proteins were purified from cell culture supernatants by capturing on nickel-loaded His-Trap chelating columns (Cytiva), and after washing until the UV absorbance returned to baseline, the protein was eluted with a 0.0–0.5 M imidazole gradient. The fractions with TGM-2 truncations were pooled and concentrated.

Mouse CD44 (NIH NCBI accession numbers XP_006498709) was expressed in HEK293 cells with coding sequences corresponding to the structurally ordered hyaluronan binding domain (aa 23–174, otherwise known as the crystallizable domain that is present in all isoforms),cloned downstream of the rat serum albumin peptide, a hexahistidine tag, and a thrombin cleavage sequence (LVPRGS) in pcDNA3.1+ (Invitrogen). Proteins were purified on HiTrap nickel chelating columns (Cytiva) and, as needed, were further polished on Superdex 75 size exclusion columns (Cytiva).

### TGF-β reporter bioassays

The fibroblast cell line MFB-F11, developed by Tesseur et al. [35] for TGF-β bioassay, tested to confirm free of mycoplasma, was cultured and the assay was performed as previously described [17]. Briefly, confluent cells were detached with trypsin and resuspended in DMEM with 2.5% FCS, 100 U/mL penicillin, 100 μg/mL streptomycin and 2 mM L-glutamine at a concentration of 8 × 10^5^ cells/mL. In 50 μL, 4 × 10^4^ cells were added to each well of a 96-well round-bottomed plate. Purified proteins, diluted at concentrations of 1–100 ng/mL were then added to each well in a volume of up to 50 μL and incubated for 24 h at 37 °C. Subsequently, 20 μL of supernatant were aspirated from each well, added to an ELISA plate (Nalge Nunc International, USA) with 180 μL of reconstituted Sigma FastTM *p*-nitrophenyl phosphate substrate and incubated at room temperature in the dark overnight. All plates were then read on an Emax precision microplate reader at 405 nm (Molecular Devices, USA). All reactions were run in duplicate and repeated at least thrice.

### pSMAD stimulation

Wild-type and CD44-KO MFB-F11 cells (previously generated using CRISPR [27]) were cultured in 6-well tissue culture plates (Corning™) until they reached a confluency of 80–90% in complete growth medium (DMEM, 10% FBS, 1% L-glutamine, 1X penicillin-streptomycin). The growth medium was then replaced with serum-free DMEM, and cells were incubated at 37 °C with 5% CO_2_ for 4 h. To stimulate SMAD2 phosphorylation, TGF-β, TGM-1, TGM-2, or truncated constructs of TGM-2 were added to the cells and incubated at 37 °C for 1 h. The cells were washed with ice-cold PBS and lysed with RIPA buffer (0.05 M Tris-HCl, pH 7.4, 0.15 M NaCl, 0.25% deoxycholic acid, 1% NP-40, 1 mM EDTA) containing 1X Halt protease and phosphatase inhibitors (Invitrogen™). Cell lysates were then cleared by centrifugation at 13,000 × *g*, 4 °C for 5 min, and protein concentrations were estimated using the Precision Red reagent.

### Foxp3^+^ Treg induction assay

Male Foxp3-GFP BALB/c mice were culled, and spleens isolated. Single-cell suspensions were prepared by macerating the spleens through a 70 µm filter and incubating for 2–5 min in red blood cell lysis buffer (Sigma). Cells were then washed and resuspended in DMEM containing HEPES (Gibco), supplemented with 2 mM L-glutamine, 100 U/mL of penicillin and 100 μg/ml of streptomycin (Gibco), 10% heat-inactivated FCS (Gibco), and 50 µM 2-mercaptoethanol (Gibco). We Used the mouse naïve CD4^+^ T cell isolation kit for isolation of cells by magnetic sorting on the Automacs system (Miltenyi, Germany) as per the manufacturer’s instructions. Isolated CD4^+^ T cells were cultured at 2 × 10^5^ per well in flat-bottomed 96-well plates (Corning, USA) with the addition of IL-2 (Miltenyi) at a final concentration of 400 U/ml. Cultured cells were stimulated with increasing concentrations (1–100 ng/mL) of TGF-β, full-length and truncated TGM-1 and TGM-2. After 72 h, cells were stained for viability, CD4, CD44, and CD103 and assessed on a Celesta flow cytometer.

### Streptavidin pulldown

Twenty µg of full-length TGM-1, TGM-2 and TGM-2 D1–3, D1–4 and D4–5 were biotinylated with EZ-Link Sulfo-NHS-LC-Biotin (Thermo Fisher Scientific) and purified using a biotin capture kit (Cytiva). For the pulldown, wild-type MFB-F11 and CD44 knock-out cells were grown at 80–90% confluency in 15 cm tissue culture dishes, washed 3× with ice-cold PBS and incubated with ~3.5 µg of biotinylated TGMs for 3 h on ice. Cells were washed 3× with ice-cold PBS and lysed with Cell Lysis Buffer (100 mM NaCl, 25 mM Tris, pH7.5, 5 mM MgCl_2_ and 0.5% NP40) supplemented with 1x Halt protease inhibitor (Thermo Scientific, 1861279) and phosphatase inhibitor (Thermo Scientific, 78427) cocktails. Cell lysates were cleared by centrifugation (13,000 × *g*, 10 min) and 2 µg of protein in each lysate was incubated with 30 µL of Neutravidin agarose beads (Thermo Scientific, 29201) for 1 h at 4 °C. Beads were washed with lysis buffer 4× (5 min each). For mass-spectrometry, beads were stored in 100 mM ammonium bicarbonate at −20 °C. For western blotting, 50 µL LDS sample buffer (Invitrogen, NP0007) containing 25 mM dithiothreitol (DTT) was added to beads and heated for 5 min at 100 °C. Denatured proteins were then run on a pre-cast polyacrylamide gel and blotted to a membrane for antibody probing.

### Mass-spectrometry (LC-MS)

Neutravidin agarose beads were resuspended in a 2 M urea and 100 mM ammonium bicarbonate buffer and stored at −20 °C. Three biological replicates for each condition were digested with Lys-C (Alpha Laboratories) and trypsin (Promega) on beads. Peptides resulting from all trypsin digestions were separated by nanoscale C18 reverse-phase liquid chromatography using an EASY-nLC II 1200 (Thermo Scientific) coupled to an Orbitrap Q-Exactive HF mass spectrometer (Thermo Scientific). Elution was carried out at a flow rate of 300 nL/min using a binary gradient, into a 20 cm fused silica emitter (New Objective) packed in-house with ReproSil-Pur C18-AQ, 1.9 μm resin (Dr Maisch HPLC GmbH), for a total run-time duration of 125 min. Packed emitter was kept at 35 °C by means of a column oven (Sonation) integrated into the nanoelectrospray ion source (Thermo Scientific). Eluting peptides were electrosprayed into the mass spectrometer using a nanoelectrospray ion source. An Active Background Ion Reduction Device (ESI Source Solutions) was used to decrease air contaminant signal levels. Xcalibur 4.2 software (Thermo Scientific) was used for data acquisition. A full scan was acquired at a resolution of 120,000 at 200 m/z, over a mass range of 350–1400 m/z. HCD fragmentation was triggered for the top 15 most intense ions detected in the full scan. Ions were isolated for fragmentation with a target of 1E5 ions, for a maximum of 125 ms, at a resolution of 15,000 at 200 m/z. Ions that had already been selected for MS2 were dynamically excluded for 20 s.

The MS Raw data were processed with MaxQuant software [36] version 1.6.14.0 and searched with Andromeda search engine [37], querying SwissProt *Mus musculus* sequences. First and main searches were performed with precursor mass tolerances of 20 ppm and 4.5 ppm, respectively, and MS/MS tolerance of 20 ppm. The minimum peptide length was set to six amino acids and specificity for trypsin cleavage was required. Cysteine carbamidomethylation was set as fixed modification, whereas Methionine oxidation, Phosphorylation on Serine-Threonine-Tyrosine, and N-terminal acetylation were specified as variable modifications. The peptide, protein, and site false discovery rate (FDR) was set to 1%. All MaxQuant outputs were analysed with Perseus software version 1.6.13.0 [38]. Protein abundance was measured using label-free quantification (LFQ) intensities reported in the ProteinGroups.txt file. Only proteins quantified in all replicates in at least one group, were measured according to the label-free quantification algorithm available in MaxQuant. Missing values were imputed separately for each column, and significantly enriched proteins were selected using a permutation-based Student’s *t* test with FDR set at 5%.

### Isothermal titration calorimetry

ITC data were generated using a Microcal PEAQ-ITC instrument (Malvern Instruments, Westborough, MA). All experiments were performed in ITC buffer (25 mM Na_2_HPO_4_, 50 mM NaCl, 0.05% NaN_3_, pH 6.0). The proteins in the syringe and sample cell and their concentrations are provided in the respective data tables. Prior to each experiment, all proteins were dialysed three times against ITC buffer and were concentrated or diluted as necessary before being loaded into the sample cell or syringe. For each experiment, thirteen 3.0 μL or nineteen 2.5 μL injections were performed, with an injection duration of 4 s, a spacing of 150 s, and a reference power of 10. Integration and data fitting were performed using Nitpic [39] and Sedphat [40]. No more than two outlier data points were removed from any one ITC data set for analysis. The binding experiments were globally fit to a simple binding model from two experimental replicates.

### In vivo *Alternaria* allergy experiments

*Alternaria* allergen was used as a model of asthma as previously described [30, 41]. Ten micrograms of *Alternaria* allergen, either alone or mixed with 1 μg of TGM-1, TGM-2 or truncated TGM-2 D1–3, were administered intranasally to mice under isoflurane anaesthesia. Animals were euthanized 24 h later to collect bronchoalveolar lavage fluid (BAL) and lung tissues for flow cytometric analysis.

### Quantification, statistical analysis and software

Western blotting was quantified using ImageJ (FIJI). FACS data were analysed with FlowJo. Data met the assumptions of a normal distribution with similar variances between groups. Unpaired Student’s *t* Test, Wilcoxon test, Fischer exact test or ANOVA statistical analyses were performed using Graph Pad Prism, as specified in each Figure Legend.

## Results

### Expression of truncated TGM-2 proteins

**Fig. 1:**
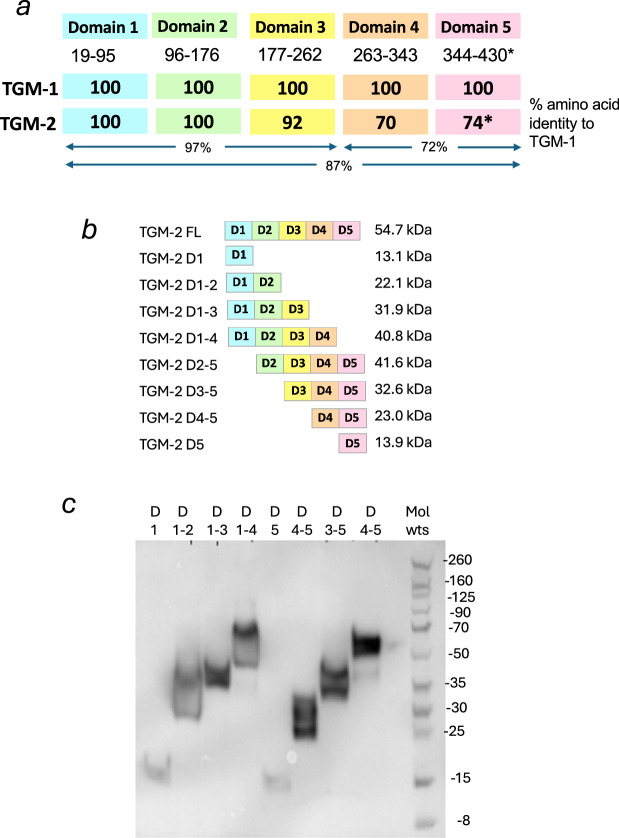
Homology and predicted structure of TGM-2. **a** Schematic of TGM-1 and TGM-2 indicating the amino acids comprising each of the 5 domains, and the percentage amino acid identity of domains to TGM-1; also indicated are identities across the TGF-β receptor-binding domains (D1–3) and the CD44-binding domains (D4–5). *Denotes that domain 5 (D5) of TGM-2 contains an 8-aa C-terminal extension as shown in the full amino acid alignment (Supplementary Fig. 1a). Note that the percent identities shown for D3-D5 are corrected with respect to those published in Smyth et al. [17]. **b** Schematic of truncation constructs of the five-domain full-length (FL) TGM-2 proteins, with molecular weights indicated. **c** Expression of each of the 8 truncated TGM-2 proteins in mammalian (Expi293) cells, revealed by Western blotting with anti- 6xHis antibody; molecular weights in kDa are indicated on the right of the panel.

Full-length *Hp*-TGM-1 and *Hp*-TGM-2 (TGM-1 and TGM-2, respectively) were previously reported to bind to TGFBR1 and TGFBR2 to activate the canonical TGF-β pathway in the MFB-F11 reporter fibroblast cell line and induce the expression of Foxp3^+^ Tregs from primary murine splenic lymphocytes [17, 42]. The parasite TGM-2 consists of five complement control protein (CCP) family domains which interact with multiple mammalian receptors. TGM-2 is closely related to TGM-1 with 100% amino acid identity for Domains 1 and 2 (D1/2), and 70–92% identity across the remaining 3 domains (Fig. 1a, Supplementary Fig. 1a), resulting in highly similar predicted 3-dimensional structures (Supplementary Fig. 1b).

To evaluate the functions of the various receptor binding domains of TGM-2, we systematically truncated both the N- and C-terminal of full-length TGM-2 to generate eight distinct recombinant protein truncations, depicted in Fig. 1b, with properties listed in Supplementary Tables 1, 2. The coding sequence for each truncation was amplified using domain-specific primers (Supplementary Tables 2, 3 Supplementary Fig. 2a, b) for cloning into the pSecTag2A mammalian expression vector that includes a C-terminal 6-histidine tag. Figure 1c presents the recombinant protein expression and secretion of all eight truncated constructs revealed by an anti-His-Tag western blot analysis of expi-293 T suspension cell culture supernatants. Recombinant proteins were purified from supernatants using His-trap nickel chelating columns on an AKTA system, with the pure proteins identified as peaks in the chromatogram. The histidine-tagged proteins were eluted from the column with a linear imidazole gradient. Each protein was analysed by Coomassie staining and wells with confirmed bands were pooled (Supplementary Fig. 2c–f), dialysed to remove imidazole, and concentrated for downstream analysis.

### Testing full-length and truncated TGM-2 proteins

**Fig. 2:**
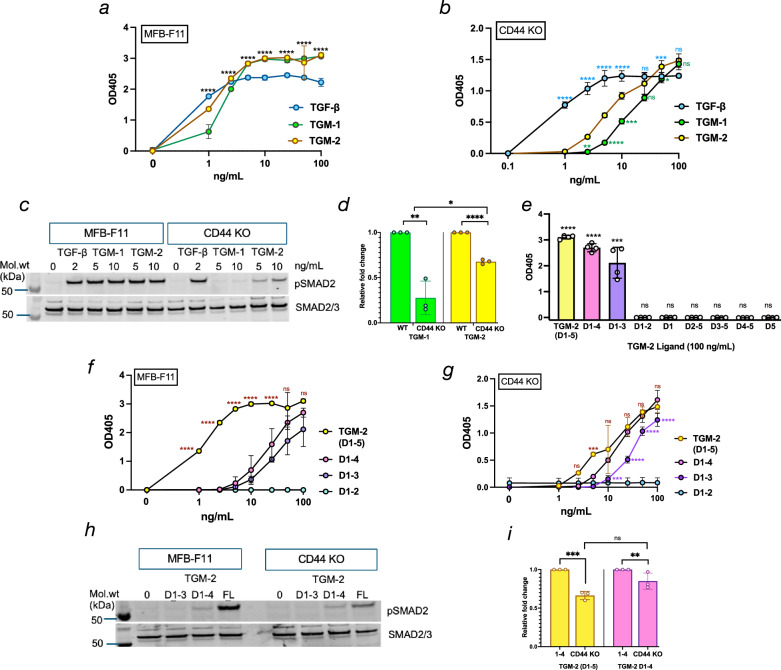
Comparison of TGF-β signalling in MFB-F11 fibroblast reporter cells by TGM-2 and truncated constructs. Full-length TGM-1, TGM-2 and each of the TGM-2 truncations were tested in MFB-F11 reporter fibroblasts that secrete alkaline phosphatase in response to SMAD phosphorylation by the TGF-β receptor kinase ALK5/TGFBR1 [35]. In some assays, CD44-deficient (KO) MFB-F11 fibroblasts [27] were also tested. **a** TGM-2 was tested at concentrations of 1–100 ng/mL, in comparison to TGM-1 and mammalian TGF-β. Data are shown as the optical density at 405 nm (OD_405_) in an alkaline phosphatase assay and are pooled from two biological replicate experiments (*n* = 4), analysed by 2-way ANOVA, with significance shown for comparisons between each TGM-2 dose and the zero control. Symbols represent means ± SD, and error bars only visible where they extend beyond the symbol size. ****p < 0.0001. **b** As (**a**) but tested on CD44- KO MFB-F11 fibroblasts; data are pooled from two biological replicate experiments (*n* = 4), analysed by 2-way ANOVA, with significance shown for comparisons between each TGM-2 dose and TGF-β (blue asterisks), and comparisons between each TGM-2 dose and TGM-1 (green asterisks). Symbols represent means ± SD, and error bars only visible where they extend beyond the symbol size. *p < 0.05; **p < 0.01; ***p < 0.001; ****p < 0.0001; ns not significant (*p* > 0.05). **c** pSMAD2 stimulation by TGM-2, compared to TGM-1 and TGF-β, evaluated by Western blot of cell lysates probed with anti-pSMAD2/3 antibodies, showing 1 of 3 replicate experiments. All 3 replicate blots are presented in Supplementary Fig. 4. **d** Densitometric analysis of all 3 replicate experiments, normalised to values from wild-type (WT) samples presented as percent relative fold change. Statistical differences between groups were determined by one-sample *t* test; *p < 0.05; **p < 0.01; ****p < 0.0001. **e** Analysis of TGM-2 truncations in wild-type MFB-F11 fibroblasts; data shown are OD405 values from samples tested at 100 ng/mL, in 3 biological replicate experiments (*n* = 6), and analysed by *t* test compared to no ligand controls; ***p < 0.001; ****p < 0.0001. Titration of activation by TGM-2 truncations in wild-type (**f**) and CD44-KO (**g**) MFB-F11 fibroblasts, showing active ligands (full length D1–5, D1–4, D1–3) and one inactive (D1–2) ligand; all other truncations tested negative but are omitted for sake of clarity. Data are shown as OD405 in an alkaline phosphatase assay and are pooled from two biological replicate experiments (*n* = 4); statistical analyses by 2-way ANOVA are shown comparing TGM-2 D1–5 with D1–4 (brown asterisks in (**f**) and (**g**)), and comparing TGM-2 D1–4 with D1–3 (purple asterisks in (**g**)). Symbols represent means ± SD, and error bars only visible where they extend beyond the symbol size. ***p < 0.001; ****p < 0.0001, ns not significant (*P* > 0.05). **h**, **i** pSMAD activation of full length (FL) TGM-2 and TGM-2 truncations, tested at 10 ng/mL, in wild-type and CD44-KO MFB-F11 cells, showing one of 3 replicate Western blots (**h**) and densitometric analysis comparing TGM-2 FL with D1–4 from all 3 replicates. Statistical differences between WT and CD44KO were determined by one-sample *t* test; **p < 0.01; ***p < 0.001.

We first tested recombinant TGM-2 protein for ability to activate signalling in MFB-F11 fibroblasts, which was found to equal or surpass the potency of TGF-β, as previously reported [17] (Fig. 2a). In parallel assays on MFB-F11 cells with CRISPR-Cas9 inactivated CD44 [27], both TGM-1 and TGM-2 showed a dramatic reduction in potency, while responses to TGF-β were unaffected (Fig. 2b). While the dose responses of wild-type MFB-F11 cells to TGM-1 and -2 were broadly similar, TGM-2 was significantly more stimulatory than TGM-1 when tested on CD44-deficient cells (Fig. 2b). SMAD phosphorylation was also evaluated by Western blotting with anti-pSMAD antibody (Fig. 2c), indicating that TGM-2 stimulation was less CD44-dependent, as confirmed by densitometric analysis of replicate Western blot assays (Fig. 2d).

We then investigated the ability of full-length recombinant TGM-2, and truncations lacking one or more receptor binding domains, to activate the TGF-β pathway. It was found that only TGM-2 constructs containing the first 3 domains (D1–3) activated TGF-β signalling in MFB-F11 cells. Other constructs lacking even one of these 3 domains, such as D1–2 and D2–5 were devoid of activity (Fig. 2e).

Our findings also revealed that TGM-2 requires domains 4 and 5 for maximum potency in wild type MFB-F11 cells as the truncated constructs D1–3 and D1–4 were an order of magnitude weaker compared to full length protein (Fig. 2f). However, in CD44 KO MFB-F11 cells, the differential effect of full length versus truncated TGM-2 was greatly reduced, with the D1–4 construct eliciting responses not significantly different from full-length D1–5 (Fig. 2g, Supplementary Fig. 3a–c). With respect to the CD44-dependence of enhanced TGF-β signaling by TGM-2, these results are similar to those observed for TGM-1 ([25] and Supplementary Fig. 3d), except for the indication that D4 alone can enhance signaling in the absence of CD44.

We also performed Western blot analyses with anti-pSMAD2 probes, confirming attenuated signaling with truncated proteins (Fig. 2h, i). There remains a residual stimulus with D1–4 that is marginally stronger than D1–3 even in CD44-deficient fibroblasts that was also evident in the MFB-F11 reporter cell assay (Fig. 2g).

### Induction of Foxp3 expression in CD4^+^ T cells

**Fig. 3:**
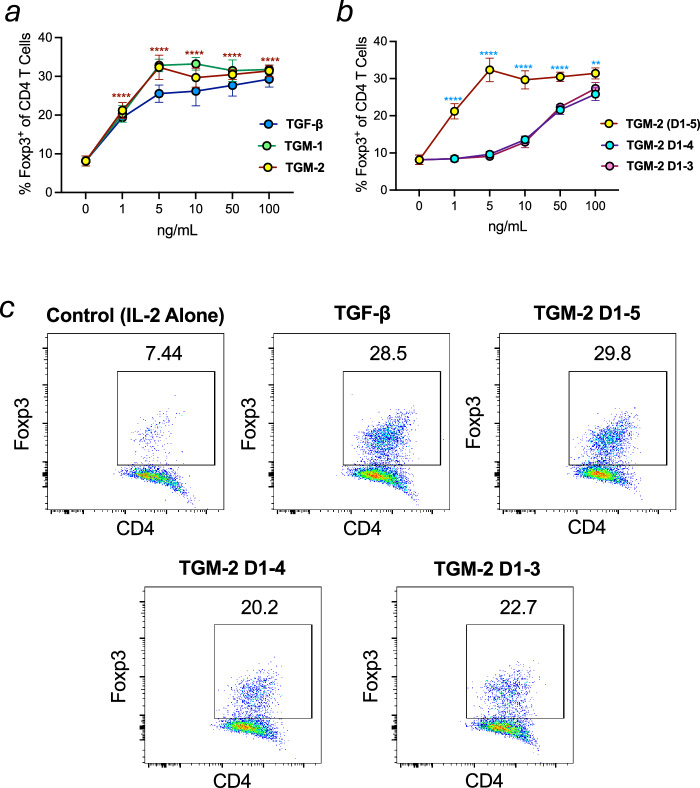
Induction of Foxp3 expression in murine CD4^+^ T cells by TGM-2 and truncations. **a**, **b** Levels of Foxp3 expression in splenic CD4^+^ T cells after culture with full length TGM-1 and TGM-2 (**a**) and TGM-2 D1–4 and TGM-2 D1–3 at concentrations of 1–100 ng/mL determined using the GraphPad prism version 10.3. Data are from one of two replicate experiments each with *n* = 3, analysed by 2-way ANOVA, for the comparisons between TGM-2 and no ligand control (brown asterisks, **a**), and between TGM-2 D1–5 and D1–4 (blue asterisks, **b**). Symbols represent means ± SD, and error bars only visible where they extend beyond the symbol size. **p < 0.01; ****p < 0.0001. **c** Representative plots showing Foxp3 expression in samples from each group. More detail on the gating strategy is given in Supplementary Fig. 5.

To further examine the functional capacity of full-length and truncated TGM-1 and TGM-2 proteins, spleens from Foxp3-GFP BALB/c mice were processed to isolate naive CD4^+^ T cells. Plated cells were stimulated with increasing concentrations of TGF-β, TGM-1, TGM-2, and truncated TGMs lacking D4 and D5, or D5 alone. After 72 h, cells were stained for viability, CD4 and assessed for Foxp3 expression by flow cytometry using the gating strategy shown in Supplementary Fig. 4. All proteins induced progressively greater numbers of Foxp3^+^ cells with increased concentrations, with full-length TGM-1 and TGM-2 inducing higher levels of iTregs relative to TGF-β (Fig. 3a–c). As with MFB-F11 cell assays, there was an approximately ten-fold loss in potency for Foxp3^+^ Treg induction in CD4^+^ cells treated with constructs lacking D4 and D5, or D5 alone, compared to full-length proteins.

### CD44 co-receptor binding by TGM-2

TGM-1 is known to bind to TGFBR2 through domain 3, which is 92% identical in TGM4 [43] (Fig. 1a). Recently domains 4 and 5 of TGM-1 were shown to bind CD44, while the corresponding domains of TGM-4 also bind CD49d and CD206 [25, 27]. However, these domains show significant divergence with TGM-2, differing in their amino acid sequence identity from TGM-1 by up to 30% (Fig. 1a). We therefore performed a binding and pull-down analysis of putative co-receptors for TGM-2, using methods similar to those recently reported in which biotin-labelled ligands are incubated with cells prior to lysis and precipitation with streptavidin beads, followed by Western blot analysis with receptor-specific antibodies [27]. As TGM-1 is known to bind CD44 [25], all analyses were performed in parallel on wildtype and CD44-deficient MFB-F11 fibroblasts.

**Fig. 4:**
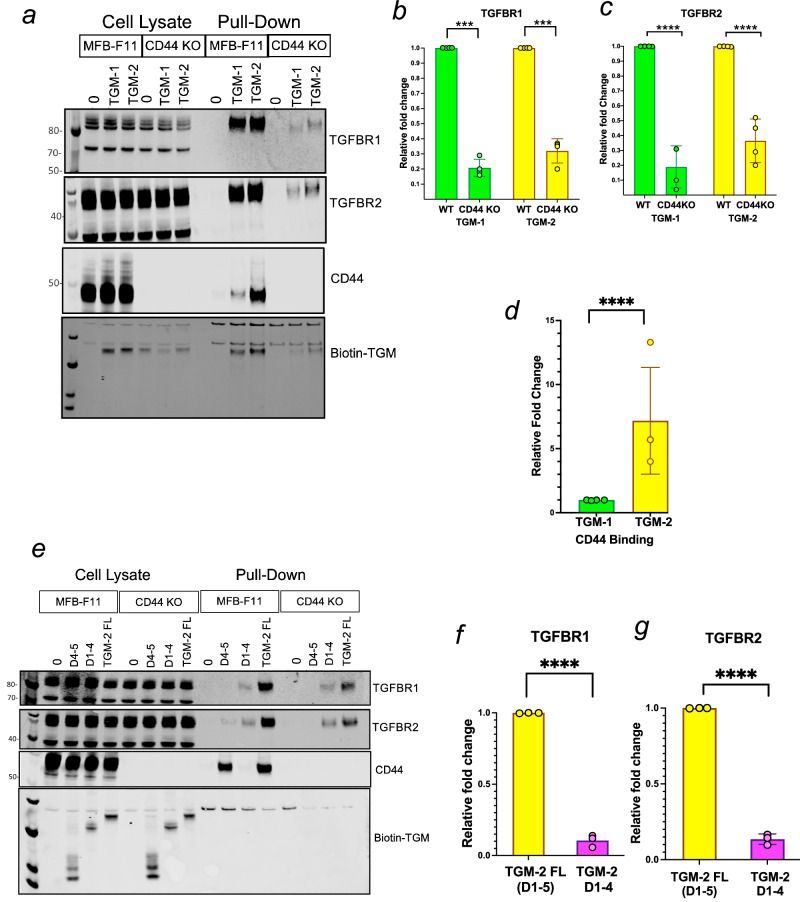
Pull-down analyses of TGM-1 and TGM-2 protein interactions with receptors. Wild type and CD44 knock out MFB-F11 cells were incubated with 20 µg of biotin-labelled recombinant full length TGM-1, TGM-2 and truncated TGM-2 D1–4 and D4–5; cell lysates pulled down with streptavidin beads were analysed by western blot. **a** Western blot analyses of anti-TGFBR1, anti-TGFBR2 and anti-CD44, from whole cell lysates and pull downs of full length TGM-1 and TGM-2. Densitometric quantification of TGFBR1 (**b**), and TGFBR2 (**c**) in pull down fractions of full length TGM-1 (green) and TGM-2 (yellow) from WT and CD44 KO cells. Statistical significance between the wild type and CD44KO for the two groups was determined by one sample *t* test. ***p < 0.001; ****p < 0.0001. **d** Densitometric quantification of CD44 pulldown fraction of full length TGM-1 and TGM-2 determined by unpaired test ***p < 0.001, **e** Western blot analysis of anti-TGFBR1, anti-TGFBR2 and anti-CD44 from whole cell lysates and pull downs of TGM-2 D1234, TGM-2 D45 and full length TGM-2. **f**, **g** Densitometric quantification of TGFBR1 and TGFBR2 pull down by TGM-2 D1234 and full length TGM-2 determined by unpaired *t* test. ****p < 0.0001.

In this manner, we confirmed that TGM-2, like TGM-1, binds to TGFBR1 and TGFBR2 (Fig. 4a), although in the absence of CD44 expression by MFB-F11 cells, the intensity of pull-down of these receptors was significantly weakened (Fig. 4b, c). Quantitatively, however, CD44 precipitation was substantially greater with TGM-2 than TGM-1 (Fig. 4d). We next tested truncated constructs of TGM-2 lacking domain 5 (D1–4) or containing only D4–5. These assays show reduced binding to TGFBR1 and TGFBR2 in the absence of D5, and that this strength of binding is not influenced by the absence of CD44, suggesting a contribution of D5 to TGFBR interactions that has not been observed with TGM-1 (Fig. 4e). This finding is also supported by densitometric analysis quantifying band area intensities from three replicate experiments, revealing a statistically significant reduction in both TGFBR1 and TGFBR2 precipitated by the truncated protein lacking D5 (Fig. 4f, g).

### Biophysical characterization of the binding affinity of TGM-1 and TGM-2 to TGFBR2 and CD44

**Fig. 5:**
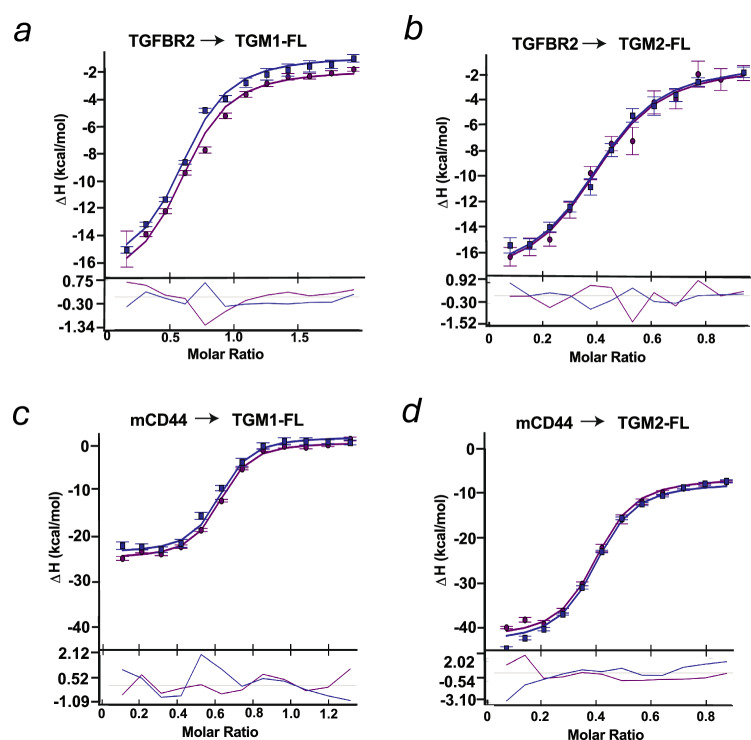
Biophysical measurement of CD44 and TGFBR2 interactions with TGM-1 and TGM-2. ITC-determined binding for recombinant TGFBR2 injected into TGM-1 FL (**a**), TGM-2 FL (**b**), or of recombinant mouse CD44 injected into TGM-1 FL (**c**), or TGM-2 FL (**d**). The binding isotherms were globally fit to a 1:1 model from two experimental replicates to yield the disassociation constants (KD) and enthalpies (see Tables 1 and 2).

As the amino acid sequences of domains 3, 4, and 5 differ between TGM-1 and TGM-2, we characterized the binding affinity of these proteins to both TGFBR2 and mouse CD44 (mCD44) by isothermal titration calorimetry (ITC), which quantitates the equilibrium disassociation constant (K_D_) in solution by measurement of the heat that is dissipated upon binding one another. Although CD44 includes multiple splice variants, we used the hyaluronan-binding domain (aa 23–174) that had previously been found to bind TGM-1 [25]. We titrated either TGFBR2 (Fig. 5a, b) or mCD44 (Fig. 5c, d) into either TGM-1 FL (Fig. 5a, b) or TGM-2 FL (Fig. 5b, d). We performed these experiments twice and we globally fit the data from both measurements to a 1:1 binding model to obtain both the K_D_s and enthalpies (ΔH) and their estimated errors (Tables 1, 2). The K_D_ of binding for the TGFBR2:TGM-1 FL interaction and the TGFBR2:TGM-2 FL interactions were 583 nM and and 403 nM, with estimated upper and lower bounds for the error of 150–300 nM, respectively (Table 1), indicating no significant difference in the binding between TGM-1 and TGM-2 with TGFBR2, which is as expected given the high sequence identity of 92% between domain 3 of TGM-1 and TGM-2 (Fig. 1a). When we titrated mCD44 into TGM-1 FL and TGM-2 FL, the K_D_ values were 72 nM and 159 nM, with estimated upper and lower bounds for the error of 25–50 nM, respectively, indicating a strong sub-micromolar binding affinity in both cases, with the small difference between them not being statistically significant (Table 2).

**Table 1:** TGM-1 and TGM-2 binding to TGFBR2 as measured by ITC.

Cell	TGM-1-FL	TGM-2-FL
Syringe	TGFBR2	TGFBR2
Cell concentration (μM)	10	10.3
Syringe concentration (μM)	100	50
Temperature (°C)	35	35
K_D_ (nM)	583 (336–1033)^a,b^	403 (237–710)^a,b^
∆H (kcal mol^−1^)	−15.7 (−18.5, −13.7)^a,b^	−16.8 (−19.7, −14.8)^a,b^
∆G (kcal mol^−1^)	−8.79^a,b^	−9.02^ab^
−T∆S (kcal mol^−1^)	6.86^a,b^	7.76^ab^
Stoichiometry (*n*)	0.59^c^	0.40^c^

^a^Uncertainty reported as 68.3% confidence interval.

^b^Global fit of two replicates.

^c^Number of sites determined by incompetent fraction value on Sedphat; set to ‘1’ for K_D_ analysis.

**Table 2:** TGM-1 and TGM-2 binding to mCD44 as measured by ITC.

Cell	TGM-1-FL	TGM-2-FL
Syringe	mCD44	mCD44
Cell concentration (μM)	6.3	9.7
Syringe concentration (μM)	48	50
Temperature (°C)	35	35
K_D_ (nM)	72 (48–106)^a,b^	141 (94, 206)^a,b^
∆H (kcal mol^−1^)	−25.7 (−27.2, −24.3)^a,b^	−35.5 (−37.7, −33.6)^a,b^
∆G (kcal mol^−1^)	−10.07^b^	−9.66^b^
-T∆S (kcal mol^−1^)	15.6^b^	25.8^b^
Stoichiometry (n)	0.65^c^	0.44^c^

^a^Uncertainty reported as 68.3% confidence interval.

^b^Global fit of two replicates.

^c^Number of sites determined by incompetent fraction value on Sedphat; set to ‘1’ for K_D_ analysis.

### Interactions of full-length recombinant TGM-1 and TGM-2 with TGFBR1, TGFBR2 and CD44 Co-receptor

**Fig. 6:**
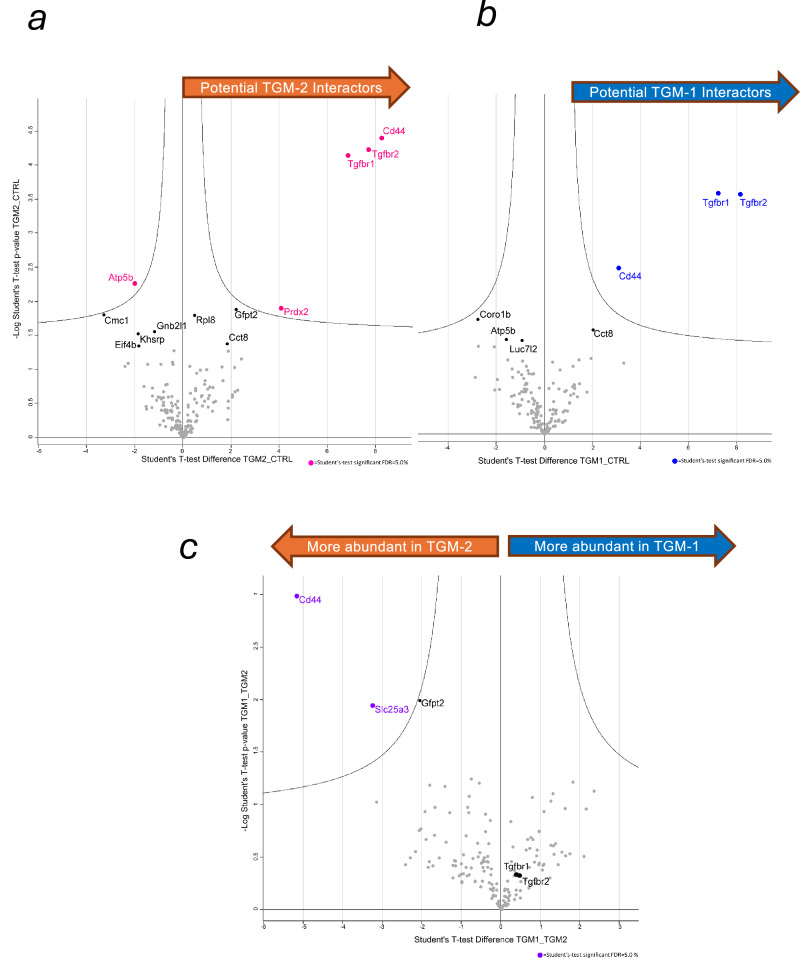
Mass spectrometric analysis of TGM-1 and TGM-2 pulldowns from cell lysates. Wild-type MFB-F11 cells were cultured and stimulated with biotin-labelled full length recombinant TGM-1 and TGM-2. Cell lysates were obtained and pulled down with streptavidin beads for MS analysis. Obtained results were statistically analysed using Perseus and represented as volcano plots. **a** Volcano plots of TGM-2/control. Magenta symbols represent candidates with false discovery rate (FDR) < 5% by Student’s *t* test. **b** Volcano plots of TGM-1/control. Blue symbols represent candidates with false discovery rate (FDR) < 5% by Student’s *t* test. **c** Volcano plots of TGM-2 /TGM-1. Green symbols represent candidates with false discovery rate (FDR) < 5% by Student’s *t* test.

To further confirm this interaction, and identify interactions of TGM-2 with other host proteins, recombinant TGM-1 and TGM-2 were labelled with biotin as previously reported [25] and a streptavidin pull-down was performed using cultured MFB-F11 cells. Triplicates of pulled-down proteins and control samples were analysed by mass spectrometry to identify interacting proteins with the results presented as volcano plots. The volcano plot in Fig. 6a reveals interaction of TGM-2 with several proteins in MFB-F11 cells, including TGFBR1, TGFBR2 and CD44 as observed with TGM-1 (Fig. 6b). Additional proteins associated with TGM2 included Prdx2, and its interactions with TGFBR1, TGFBR2 and CD44 received a higher statistical value compared to TGM-1. However, unlike CD44 which were able to confirm by Western blot analysis using anti-CD44 antibodies, we were not able to confirm the interaction of TGM-2 with Prdx2 using anti-peroxiredoxin 2 antibodies.

### Full-length TGM-1, TGM-2, and recombinant truncated Domains 1–3 of TGM-2 suppress type 2 eosinophilia in vivo

**Fig. 7:**
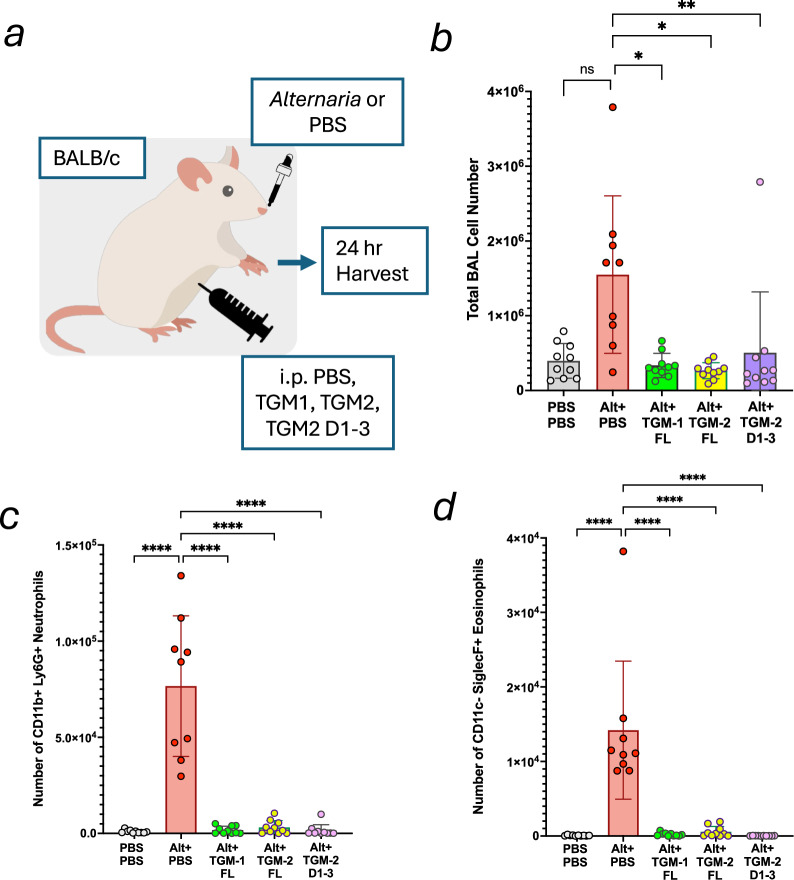
Full length TGM-1, TGM-2 and truncated TGM-2 D1–3 reduce in vivo induced allergic airway inflammation. Ten micrograms of *Alternaria* allergen, either alone or mixed with 1 μg of TGM-1, TGM-2 or TGM-2 D1–3, were administered intranasally to mice, which were euthanized 24 h later to collect bronchoalveolar lavage fluid (BAL) for flow cytometric analysis using the gating strategy shown in Supplementary Fig. 6. **a** Schematic of the experimental design. **b** Total cell counts in BAL from control mice and those exposed to *Alternaria* allergen (Alt), or *Alternaria* combined with TGM-1, TGM-2, or TGM-2 D1–3. Data are pooled from two biological replicate experiments, each with *n* = 4 or 5 and analysed by one-way ANOVA. Neutrophil (**c**) and eosinophil (**d**) counts from the same experiments. Data were analysed by one-way ANOVA with Dunnett’s multiple comparison test. *p < 0.05; ***p < 0.001; ****p < 0.0001.

The exposure of mice to the fungal model *Alternaria alternata* induces eosinophilia within 24 h of treatment [44]. Previous investigations of the role of TGM-1, and *H. polygyrus* HES, in reducing inflammation revealed potent inhibition of eosinophilic inflammation by the parasite proteins [30, 33]. In this model, we investigated the ability of full-length TGM-2 and truncated D1–3 recombinant proteins, in comparison to TGM-1, to influence the pro-allergic response to *A. alternata* 24 h after intranasal administration (Fig. 7a). We also compared the degree of suppression of induced inflammation by full-length TGM-1, TGM-2 and TGM-2 D1–3. Flow cytometric analysis of the differential cell response was analysed using the gating strategy in Supplementary Fig. 6. The total number of inflammatory cell infiltrates in the bronchoalveolar lavage fluid (BAL) was reduced by each of the TGM proteins close to the level in control mice which had not received the allergen (Fig. 7b). There was similarly a dramatic decrease in the number of eosinophils in the BAL for mice groups treated with TGM-1, TGM-2 and TGM-2 D1–3 relative to the negative control group that received *Alternaria* with PBS (Fig. 7c). Likewise, the number of neutrophils among CD45^+^ BAL cells was closer to baseline in the mice group treated with TGM-1, TGM-2 and TGM-2-D1–3 relative to the *Alternaria* with PBS group (Fig. 7d).

## Discussion

The co-evolution of parasites and their hosts can be described as an “arms race” in which both sides have developed sophisticated attack and defense mechanisms [45]. Despite the robust nature and exceptional cooperation of the immune, nervous and endocrine systems in mammals, there is often a failure to eradicate parasitic worms [46]. Rather than any inadequacy of the host immune system, susceptibility may arise from active immune manipulation and diversion by parasitic helminths towards a more immunoregulatory state, for example through Tregs [5]. There may also be advantageous consequences of such immune down-regulation on many inflammatory conditions [47]. This is evident in the contrasting difference in the prevalence of autoimmune diseases in the industrialized countries where helminths have been eradicated and the developing countries with ongoing transmission [33]. This spurred the trial of live helminths as potential therapeutic options for the treatment of inflammatory disorders [48–50], and more recently interest in defined helminth secreted molecules as future pharmacological products [9, 51]. In this context, a major breakthrough was the identification of a novel Treg-inducing protein, TGM, a mimic of the mammalian immunosuppressive cytokine TGF-β that recapitulates its function through an unrelated structure [16], and the discovery of a larger family of 10 homologous protein expressed by this parasite [17, 18].

TGM-2, the second *H. polygyrus* secreted TGF-β mimic homologue, is a 5-domain protein, previously lacked characterization beyond its ability to activate fibroblasts and T cells [17]. In this study, we first analysed the domain(s) required for TGF-β signalling by generating a series of eight truncated constructs lacking one or more domains. Our evaluation of these constructs ascertained that, as with TGM-1, domains 1–3 are essential for its TGF-β-like activity, as measured both by a fibroblast reporter cell line (MFB-F11) and by murine T cell induction of Foxp3. Furthermore, we validated the role of D1–3 by pull-down experiments in which these domains were shown to directly bind the TGFBR1 and TGFBR2 receptors. We investigated the direct affinity of TGM-2 for TGFBR2 by ITC, but the affinity was found to not significantly differ from TGM-1, consistent with the absence of any substitutions in TGM-2 D3 relative to those in TGM-1 D3 in the receptor binding interface [52]. We did not perform a similar comparison for TGFBR1 binding since the two proteins are 100% identical within domains 1.

In the absence of domains 4 or 5, TGM-2 was significantly less potent in both MFB-F11 fibroblasts and T cells, as previously found for TGM-1 in which these domains bind the co-receptor, CD44 [25]. We analysed the role of CD44 in TGM-2 activation both in CD44-deficient cells, and truncated TGM-2 proteins lacking D4 and/or D5, and performed pull-down experiments with biotinylated TGM-2 proteins. These results confirmed that D4–5 binds CD44, although quantitation revealed some differences relative to TGM-1; for example, in CD44-deficient cells, TGM-2 was found to retain greater potency for MFB-F11 activation than for TGM-1 (Fig. 2b). We therefore investigated whether TGM-2 might bind an additional co-receptor that was not recognized by TGM-1, and although the mass spectrometry pull-down measurements indicated that the anti-oxidant enzyme peroxiredoxin-2 (Prdx2) might specifically interacted with TGM-2, we were not able to confirm this by western blot. Prdx2 attenuates STAT3-dependent cytokine signaling [53], but is not known to intersect canonical TGF-β signalling, thus further investigations are necessary to study if this co-receptor might interact with and contribute to the function of TGM-2.

In these experiments, we also found that biotinylated TGM-2 showed a stronger interaction with the co-receptor CD44 than did TGM-1, while ITC measurements indicated a slightly lower direct affinity of TGM-2 for CD44. This may indicate that TGM-2 engages additional factors that raise its overall avidity, through an unidentified co-receptor, homodimerisation or unexpected participation of domains other than domains 4 and 5 in binding CD44. In this setting, further investigation into the possible role of Prdx2 as a co-receptor should be undertaken. It may also be the case that technical issues such as degree or location of biotin conjugation to lysine side chains differentially affect the affinity of TGM-1 and TGM-2 for CD44 in pulldown experiments, as we noted that across domains 4 and 5 which bind CD44, there are 6 lysine residues in TGM-1 that are substituted with other amino acids in TGM-2. Future studies could address this by employing BirA-mediated N-terminal biotinylation that does not alter ε amino side chains of lysine residues.

Aside from these more subtle differences in binding profiles of TGM-1 and TGM-2, the two homologues showed a high degree of functional similarity in immunological assays in vitro and in vivo. As reported previously [17], TGM-2 is able to induce Foxp3^+^ Tre*g* differentiation from naïve murine splenic T cells in vitro, and we further show here that the efficacy for Treg induction is highly dependent on the CD44-binding domains 4 and 5. Our data do not extend to demonstrating functional immune suppression by TGM-2-induced Tregs, as we have previously shown for TGM-1-induced Tregs [26, 42], and this remains to be ascertained.

We also tested TGM-2 in an in vivo model of airway inflammation driven by *Alternaria* fungal allergen, which reflects short-term acute innate immune cell activation in a 24-h window. TGM-2 was found to be equally effective in this setting as was previously found for TGM-1 [30]. Moreover, the truncated form of TGM-2 containing D1–3 without the CD44-binding domains proved to be equally efficacious in this model as the full-length protein; this may reflect the short time-frame of the assay in which CD44 may not be so prominent, or result from the proteins administered being above a critical threshold for immune modulation. It will be important in future work to explore whether both forms of TGM-2 are also as suppressive as TGM-1 in more extensive models of airway allergy that involve sensitized T cells, and to evaluate mechanistically whether expansion of Tregs underpins modulation of the broader, adaptive allergic response. In addition, it will be interesting to explore the effects of TGM-2 on innate effector cell types such as eosinophils, mast cells and neutrophils that are recruited at later phases of allergic inflammation, as well as innate lymphoid cells which respond through the IL-33 receptor, IL-2, that is upregulated by TGF-β [54].

As recently reviewed [18] the interplay between TGM domains and host surface receptors presents a complex pattern of multi-valent ligands and binding to multiple host cell surface receptors. The expansion of the TGM gene family, which appears to have occurred within the more recent lineage of *Heligmosomoides* [55], has resulted in homologues (TGM-1, TGM-4) with differing affinity for host TGF-β receptors, differing specificity for co-receptors, and even antagonistic activity in one member (TGM-6) which lacks the TGFBR1-binding domains D1 and D2 [25, 27, 52]. The differences between TGM-1 and TGM-2 are more modest than those between TGM-1 and TGM-4 [27] and between TGM-1 and TGM-6 [52], but nevertheless illuminate key features of receptor and co-receptor interactions that will help develop an optimal immune-regulatory mediator with therapeutic potential.

## Supplementary information

Supplemental Material

## Data Availability

The amino acid sequence of *H. polygyrus* TGM2 is deposited under the accession number AVN88293. All data analysed during this study are presented in the published article and supplementary information; primary data from the individual experiments are also available from the corresponding author upon reasonable request.
