# Supplementary material for: Structural and functional analysis of the TGF-β mimic, TGM-2: an immunomodulatory helminth protein
Source: Genes Immun. 2025 Dec 18;27(1):117–29. doi: 10.1038/s41435-025-00372-0 (PMC12923355; doi:10.1038/s41435-025-00372-0)
Supplement: Supplementary file 1 — Supplemental Material [file 41435_2025_372_MOESM1_ESM.pdf]

Structural and Functional Analysis of the TGF-β Mimic, TGM-2: an Immunomodulatory Helminth Protein

Emmaculate Yaah Ntang[1,2], Kyle T. Cunningham[1,†], Shashi P. Singh[1,¶], Claire Ciancia[1], Anna Sanders[1], Sergio Lilla[3], Ananya Mukundan[4], Stephen M. Ghogomu[2], Andrew P. Hinck[4] and Rick M. Maizels [1,*]

Supplementary Material

Supplementary Figure 1: Comparisons of TGM-1 and TGM-2 from *H. polygyrus.*

(a) Amino acid alignment of the 5 domains of TGM-1 and TGM-2. D1 and D2 share 100% identity. Identical residues are indicated in reverse shading, similar residues in grey, and dissimilar residues in white. Potential *N*-linked glycosylation sites are shown as green lollipops, and residues known to contact TGFBR2 are indicated with red arrows. A purple star is placed at an unusual Gly-to-Cys substitution.

(b) Alpha fold predicted structures of TGM-1 and TGM-2.

Supplementary Figure 2: Truncation constructs of TGM-2.

(a, b) Electropherogram of amplified inserts for plasmid cloning of truncated TGM-2 in pSecTag, showing eight individual constructs of TGM-2 lacking one to four domains from PCR amplified N (a) and C (b) terminals.

(c, d) Sample Coomassie staining and corresponding anti-His-Tag Western Blot analysis of two of the purified TGM-2 truncated recombinant proteins.

Supplementary Figure 3: Requirement for CD44 for optimal cell activation.

Comparison of activation of MFB-F11 reporter fibroblasts (34), and cells deficient in CD44 (26), measured by release of alkaline phosphatase into cell media. Data are from the same experiments as Figure 2 a, b, replotted to compare sufficient and deficient cell lines. Note that there is a general depression of reporter response in CD44 KO cells that is evident by comparing TGF-β responses in Figures 2 and b.

(a-c) TGM-2 and each of the active TGM-2 truncations.

(d) TGM-1.

Supplementary Figure 4: Replicate Western Blots; Experiment 1 is also shown in Figure 4 A. Densitometry data plotted in **Figure 4 b-d are those** measured from all 3 experiments.

Supplementary Figure 5: Gating strategy for Foxp3$^+$ Treg cells from mouse splenocyte cultures

(a) Cells are gated based on time to exclude debris

(b, c) Forward and side scatter allows doublets to be identified and excluded.

(d) Use of the fixable dead cell exclusion dye allows dead cells to be excluded
(e) Gated $CD4^+$ T cells with forward scatter.
(f) Gating for Tregs using the non-orthogonal CD4/Foxp3-GFP gate.

Supplementary Figure 6. Gating strategy for eosinophils and neutrophils

(a) Cells are gated to exclude beads
(b, c) Use of forward and side scatter to select singlets and live cells.
(d) Gating for CD45 using the SSC-A/CD45 gate
(e), (g), (i), (j) and (k) Gated Fluorescent Minus One (FMO) sample
(f) Gating for eosinophils
(h) Gating for neutrophils

Suppl. Figure 1

a

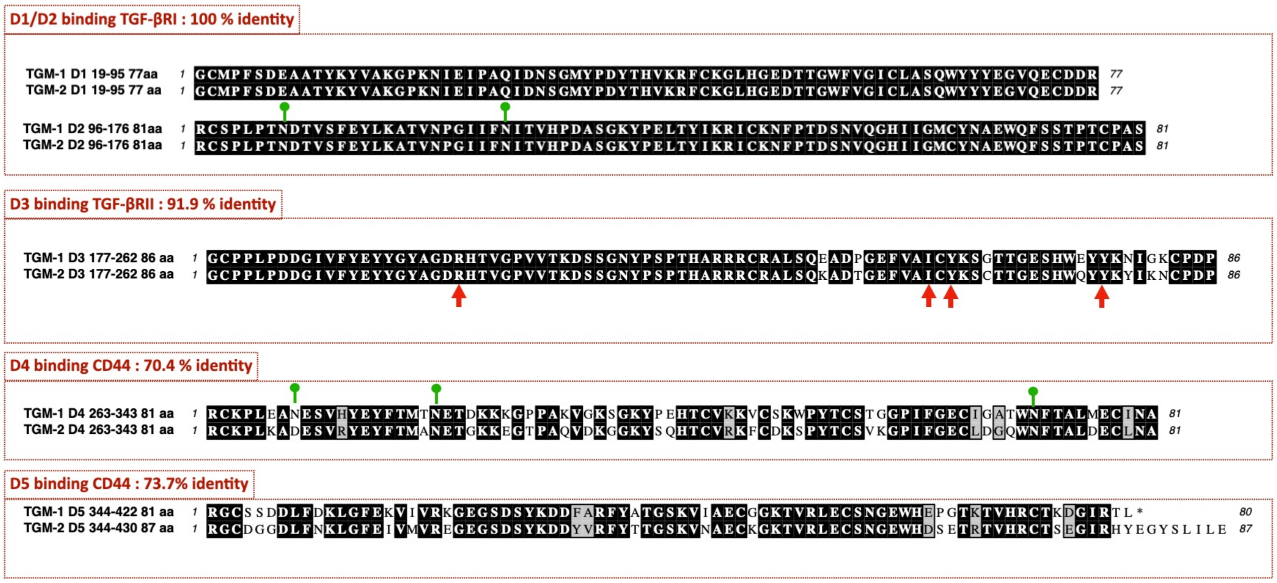

b

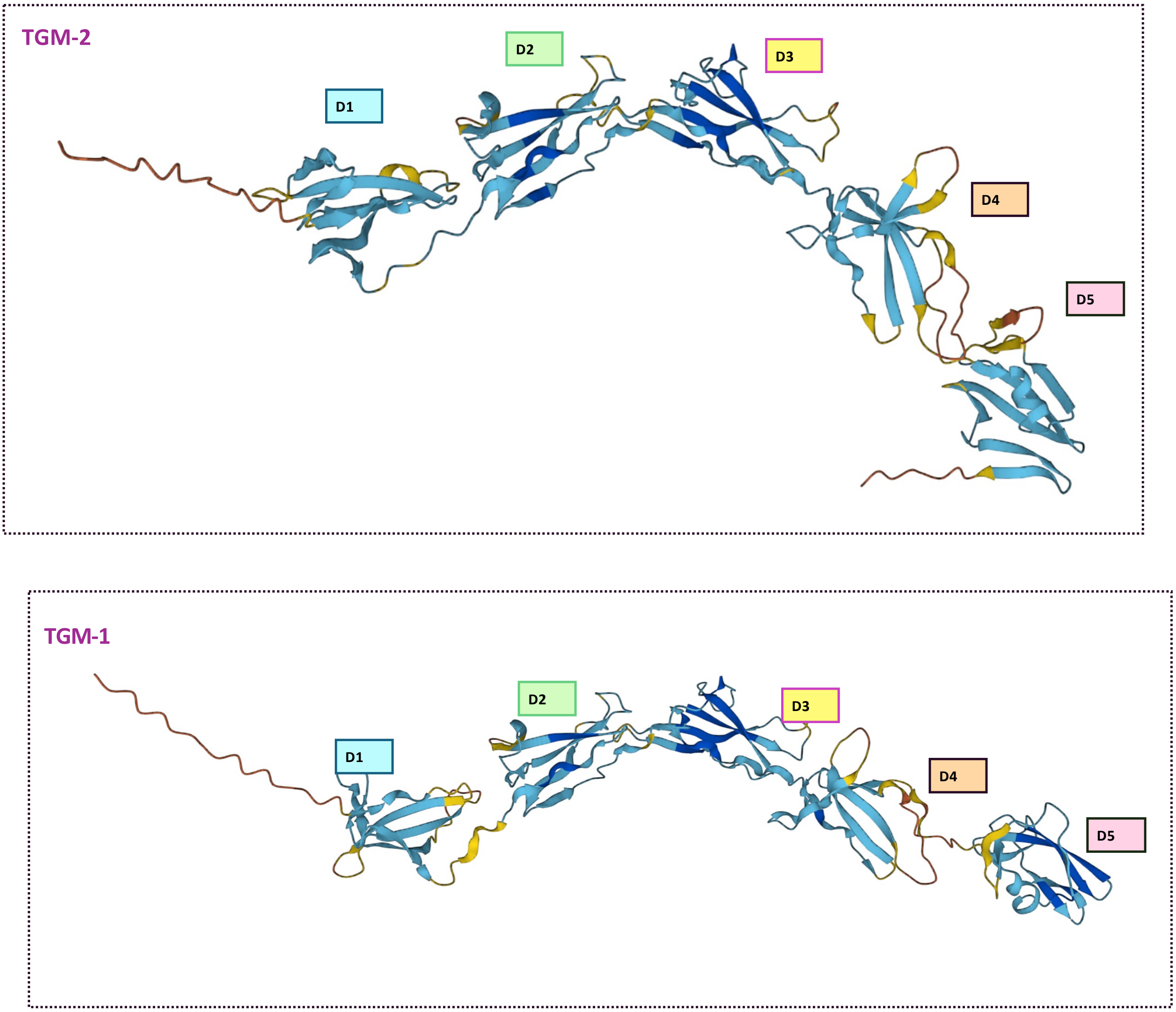

Suppl. Figure 2

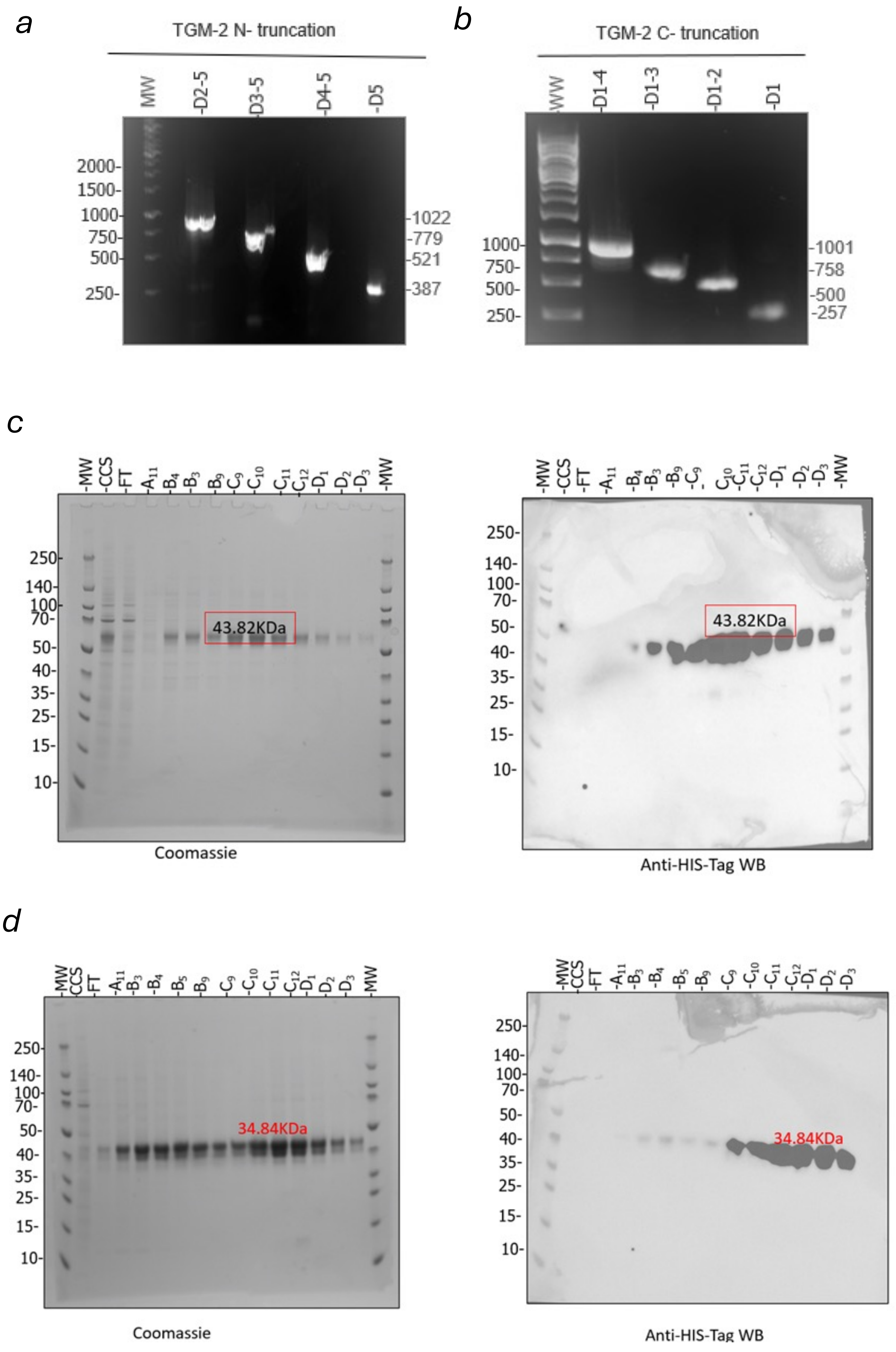

Suppl. Figure 3

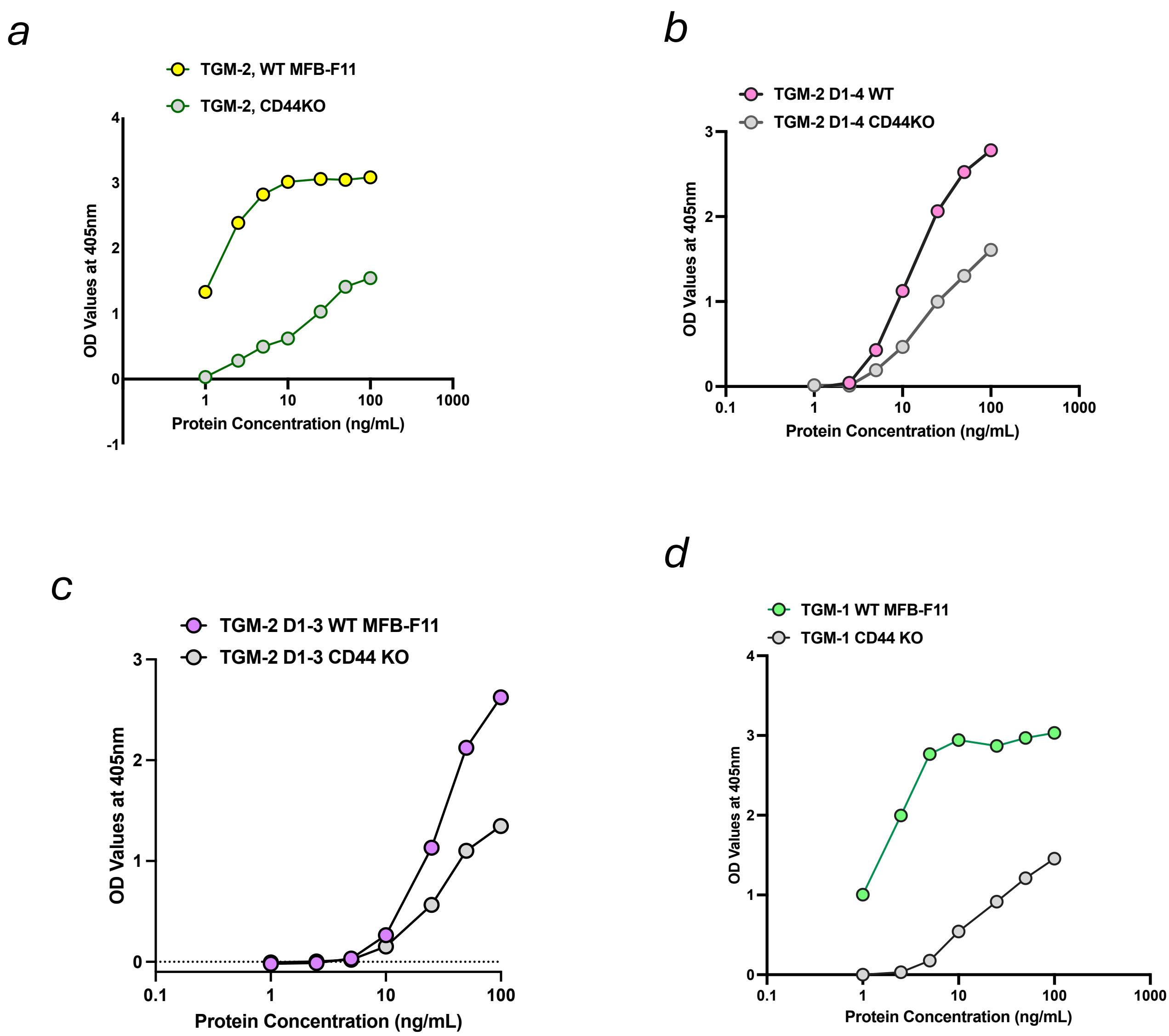

Suppl. Figure 4

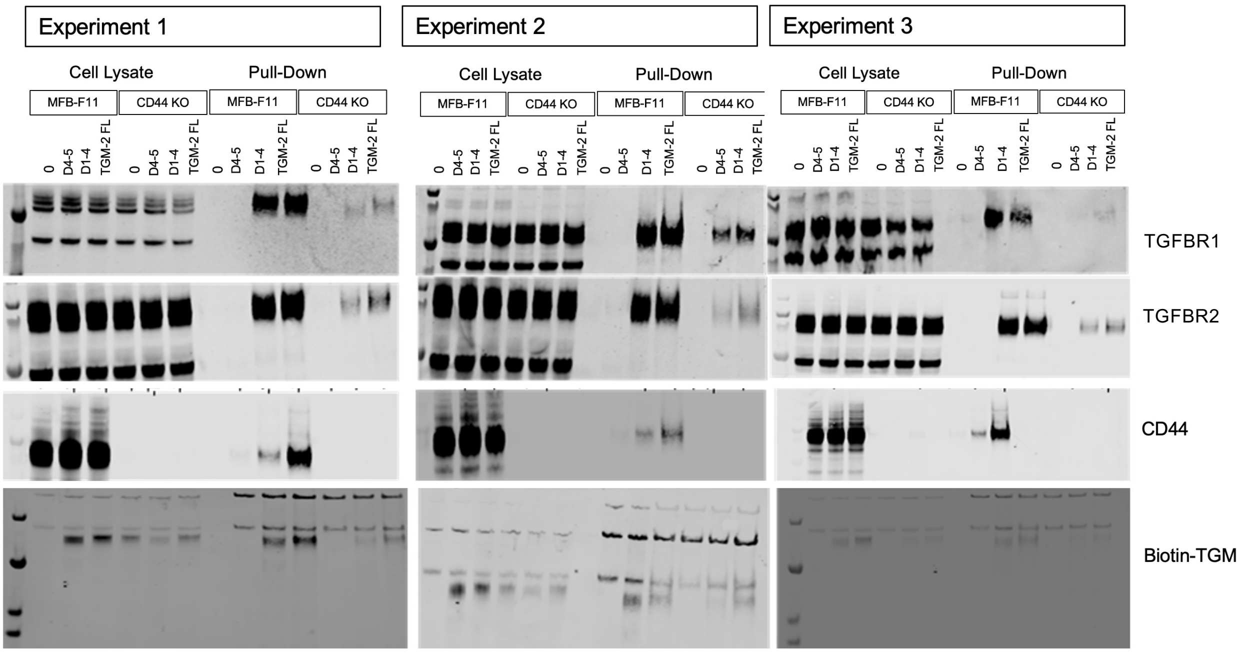

Suppl. Figure 5

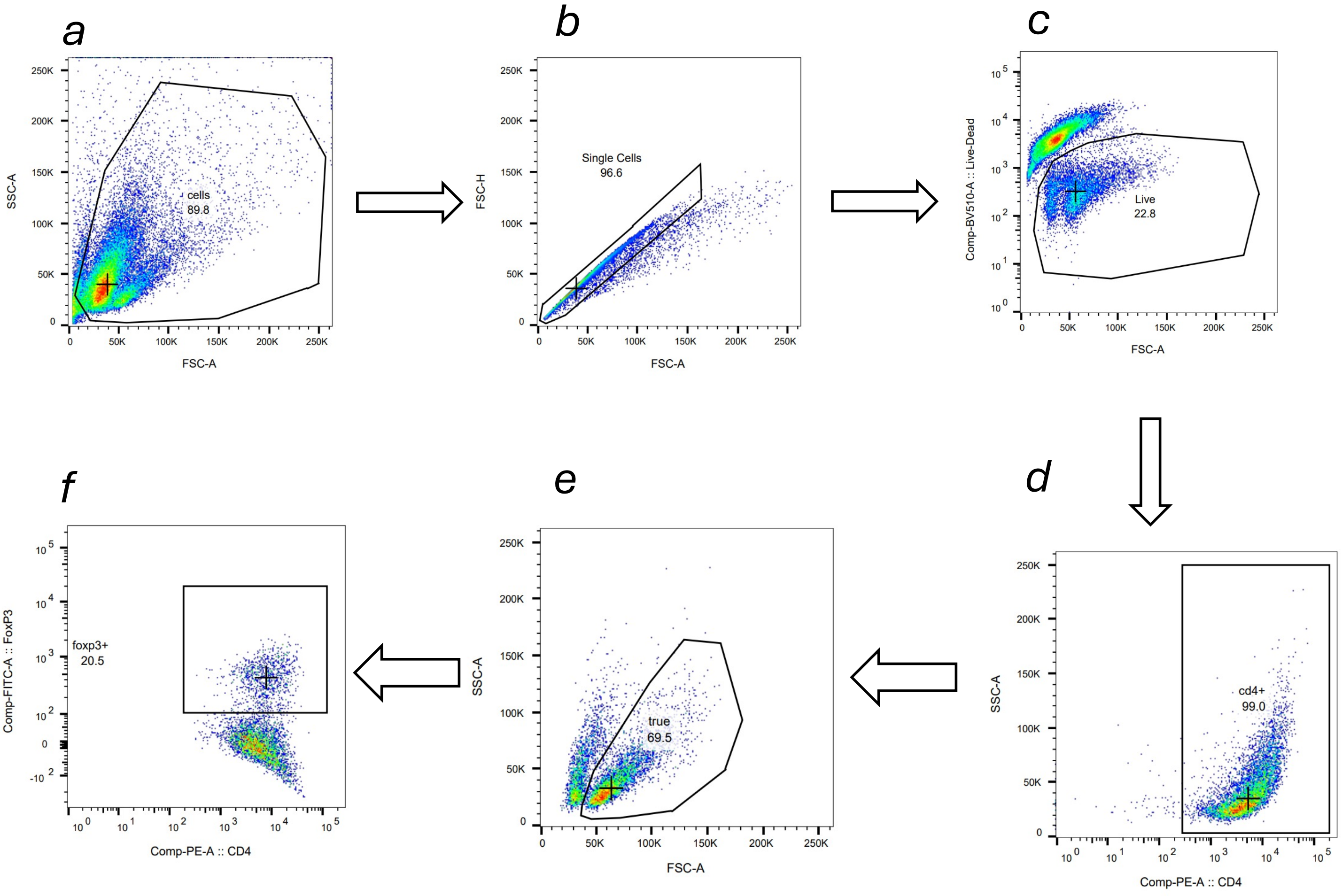

Suppl. Figure 6

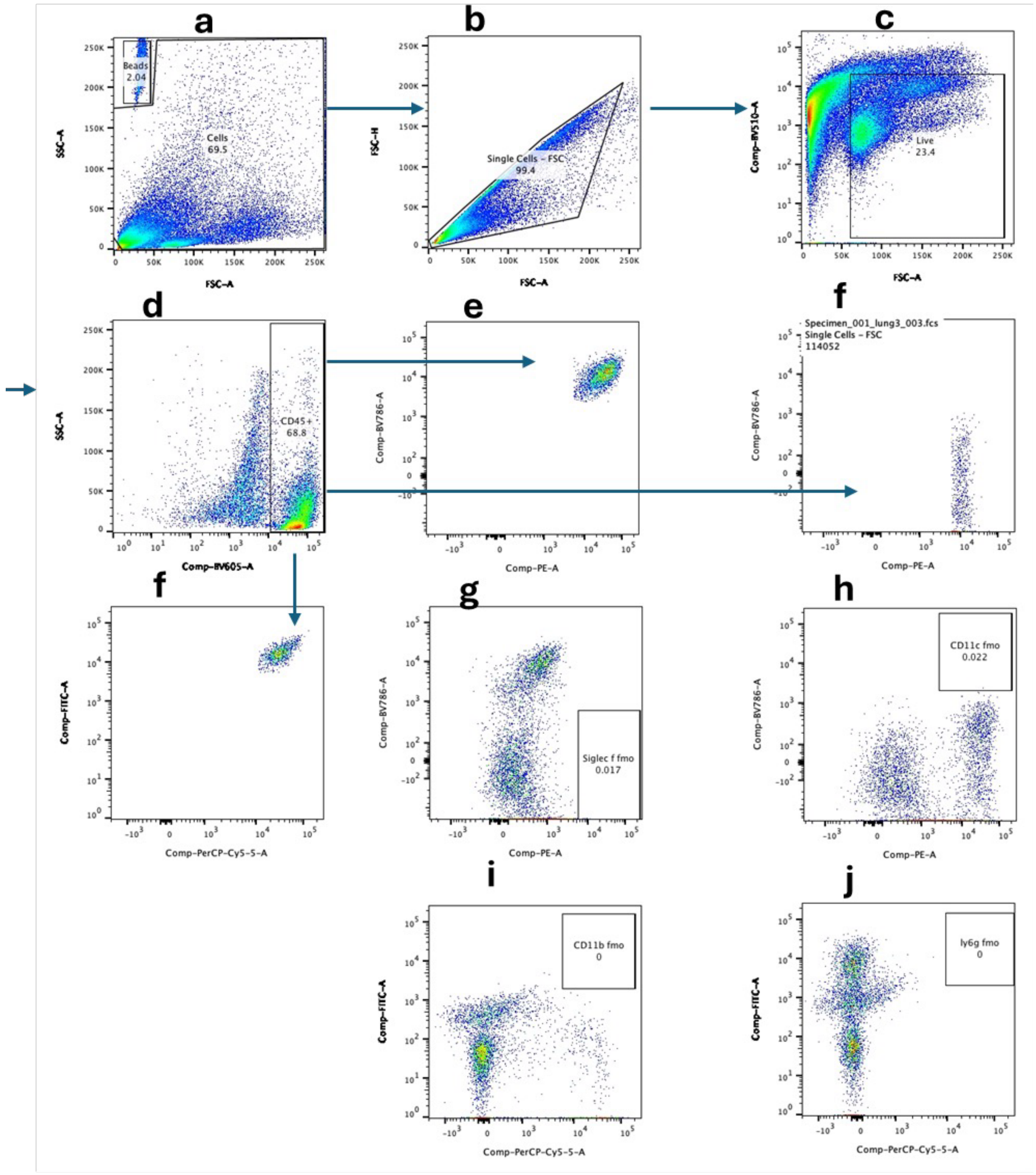

Supplementary Table 1

Nucleotide sequence of *Heligmosomoides polygyrus* Transforming Growth factor-β Mimic (TGM)-2, amino acids 16-430, codon optimized for expression in human HEK293T cells. Inserts were synthesized by GeneArt with flanking sequences for cloning into the pSecTag2A plasmid vector, downstream of a 5' vector-encoded ATG start codon and Ig Kappa chain signal peptide prior to an *Asc*I restriction site (GG^CGCGCC). The 3' end of the synthetic insert contained an *Apa*I restriction site (GGGCC^C) for in-frame insertion reading through to Myc and 6-His tags, before a TAA stop codon. The sequence below represents the inserted TGM-2 coding sequence between the two restriction sites. In the native gene, a TGA codon is found immediately after the final GAA codon.

Gene Name	Sequence of Protein Optimized	Nucleotide Sequence
**TGM-2**	16-430<br>(1245 bp)	GATGATAGCGGCTGCATGCCTTTTAGCGACGAGGCCGCCACCT<br>ACAAATACGTGGCCAAGGGCCCCAAGAACATCGAGATCCCCGC<br>CCAGATCGACAACAGCGGCATGTACCCCGACTACACCCACGTG<br>AAGCGGTTCTGCAAGGGCCTGCACGGCGAGGATACCACCGGAT<br>GGTTCGTGGGCATCTGCCTGGCCAGCCAGTGGTACTACTACGA<br>GGGCGTGCAGGAATGCGACGACAGAAGATGCAGCCCCCTGCCC<br>ACCAACGACACCGTGTCCTTCGAGTACCTGAAGGCCACCGTGA<br>ACCCCGGCATCATCTTCAACATCACCGTGCACCCTGACGCCTC<br>CGGCAAGTACCCTGAGCTGACCTACATCAAGAGGATCTGCAAG<br>AACTTCCCCACCGACAGCAACGTGCAGGGCCACATCATCGGCA<br>TGTGCTACAACGCCGAGTGGCAGTTCAGCAGCACCCCTACCTG<br>TCCTGCCAGCGGCTGTCCTCCCCTGCCTGATGATGGCATCGTG<br>TTCTACGAGTACTACGGCTACGCCGGCGACAGACACACAGTGG<br>GCCCTGTCGTGACCAAGGACAGCAGCGGCAACTACCCCAGCCC<br>TACACATGCCAGACGCCGGTGTAGAGCCCTGAGCCAGAAAGCC<br>GATACCGGCGAGTTTGTGGCCATCTGCTACAAGAGCTGCACCA<br>CCGGCGAGAGCCACTGGCAGTACTACAAGTATATCAAGAACTG<br>CCCCGACCCCCGGTGCAAGCCTCTGAAGGCCGATGAGTCCGTC<br>AGATACGAGTATTTCACCATGGCCAACGAGACAGGCAAGAAAG<br>AGGGCACACCCGCCCAGGTGGACAAGGGCGGCAAGTATAGCCA<br>GCACACCTGTGTGCGGAAGTTCTGCGACAAGTCCCCCTACACC<br>TGTAGCGTGAAGGGCCCTATCTTCGGCGAGTGCCTGGATGGCC<br>AGTGGAACTTCACCGCCCTGGATGAGTGCCTGAACGCCAGAGG<br>ATGTGACGGCGGCGACCTGTTCAACAAGCTGGGCTTCGAGATC<br>GTGATGGTGCGCGAGGGCGAGGGCAGCGACAGCTACAAGGATG<br>ACTACGTGCGGTTCTACACAACCGGCAGCAAAGTGAATGCCGA<br>GTGCAAGGGCAAGACCGTGCGGCTGGAATGCAGCAATGGCGAG<br>TGGCACGACTCCGAGACACGGACCGTGCACAGATGTACCAGCG<br>AGGGCATCCGGCACTATGAGGGCTACAGCCTGATCCTGGAA

Supplementary Table 2

Systematic truncation of TGM-2 from the 5'and 3' direction.

Proteins	AA	MW	$E^{1\%}$
TGM-2 Domains 2345 (Δ1)	372	41596.35	13.44
TGM-2 Domains 345 (Δ12)	291	32616.16	13.55
TGM-2 Domains 45 (Δ123)	205	22976.16	10.90
TGM-2 Domain 5 (Δ1245)	124	13902.18	9.49
TGM-2 Domains 1234 (Δ5)	365	40790.52	15.78
TGM-2 Domains 123 (Δ54)	284	31716.26	16.57
TGM-2 Domains 12 (Δ543)	198	220776.54	15.12
TGM-2 Domain 1 (Δ5432)	117	13096.37	16.55

Supplementary Table 3

Codon optimized 5’ (a) and 3’ (b) truncation primers with *Asc1* and *Not1 restriction sites* (Capitalized) respectively

(a)

**Primer name**	**(Asc1) 5`-3`**	**Nucleotide position**	**Amino acid position**
coTGM2_domain1F	**gactGGCGCGCC**gat gat agc ggc tg c atg	46-63	16-21 DDSGCM
coTGM2_domain2F	**gactGGCGCGCC**aga tgc agc ccc ct g ccc	286-303	96-101 RCSPLP
coTGM2_domain3F	**gactGGCGCGCC**ggc tgt cct ccc ct g cct	529-547	177-182 GCPPLP
coTGM2_domain4F	**gactGGCGCGCC**cgg tgc aag cct ct g aag	787-805	263-268 RCKPLK
coTGM2_domain5F	**gactGGCGCGCC**aga gga tgt gac g gc ggc	1030-1048	344-349 RGCDGG

(b)

**Primer name**	**(Not1) 3`-5`**	**Nucleotide position**	**Amino acid position**
coTGM2_domain1R_NotI	**gactGCGGCCGC**tct gtcgtcgcattcctgcac	285-265	95-89 VQECDDR
coTGM2_domain2R_NotI	**gactGCGGCCGC**gctgg caggacaggt	528-512	176-172 TCPAS
coTGM2_domain3R_NotI	**gactGCGGCCGC**gggg tcggggcagttctt	786-769	262-257 KNCPDP
coTGM2_domain4R_NotI	**gactGCGGCCGC**ggcgt t cag gcactcatc cag	1029-1009	343-337 LDECLNA
coTGM2_domain5R_NotI	**gactGCGGCCGC**ttc cag gat cag gct	1290-1278	430-426 SLILE
